# Associations between Disaster Exposures, Peritraumatic Distress, and Posttraumatic Stress Responses in Fukushima Nuclear Plant Workers following the 2011 Nuclear Accident: The Fukushima NEWS Project Study

**DOI:** 10.1371/journal.pone.0087516

**Published:** 2014-02-19

**Authors:** Jun Shigemura, Takeshi Tanigawa, Daisuke Nishi, Yutaka Matsuoka, Soichiro Nomura, Aihide Yoshino

**Affiliations:** 1 Department of Psychiatry, National Defense Medical College, Tokorozawa, Saitama, Japan; 2 Department of Public Health, Ehime University Graduate School of Medicine, Toon, Ehime, Japan; 3 Department of Mental Health Policy and Evaluation, National Institute of Mental Health, National Center of Neurology and Psychiatry, Kodaira, Tokyo, Japan; 4 Department of Clinical Epidemiology, Translational Medical Center, National Center of Neurology and Psychiatry, Kodaira, Tokyo, Japan; 5 Department of Psychiatry, National Disaster Medical Center, Tachikawa, Tokyo, Japan; Chiba University Center for Forensic Mental Health, Japan

## Abstract

**Background:**

The 2011 Fukushima Daiichi Nuclear Power Plant accident was the worst nuclear disaster since Chernobyl. The nearby Daini plant also experienced substantial damage but remained intact. Workers for the both plants experienced multiple stressors as disaster victims and workers, as well as the criticism from the public due to their company's post-disaster management. Little is known about the psychological pathway mechanism from nuclear disaster exposures, distress during and immediately after the event (peritraumatic distress; PD), to posttraumatic stress responses (PTSR).

**Methods:**

A self-report questionnaire was administered to 1,411 plant employees (Daiichi, *n* = 831; Daini, *n = *580) 2–3 months post-disaster (total response rate: 80.2%). The socio-demographic characteristics and disaster-related experiences were assessed as independent variables. PD and PTSR were measured by the Japanese versions of Peritraumatic Distress Inventory and the Impact of Event Scale-Revised, respectively. The analysis was conducted separately for the two groups. Bivariate regression analyses were performed to assess the relationships between independent variables, PD, and PTSR. Significant variables were subsequently entered in the multiple regression analyses to explore the pathway mechanism for development of PTSR.

**Results:**

For both groups, PTSR highly associated with PD (Daiichi: adjusted β, 0.66; *p*<0.001; vs. Daini: adjusted β, 0.67; *p*<0.001). PTSR also associated with discrimination/slurs experience (Daiichi: 0.11; *p*<0.001; vs. Daini, 0.09; *p = *0.005) and presence of preexisting illness(es) (Daiichi: 0.07; *p* = 0.005; vs. Daini: 0.15; *p*<.0001). Other disaster-related variables were likely to be associated with PD than PTSR.

**Conclusion:**

Among the Fukushima nuclear plant workers, disaster exposures associated with PD. PTSR was highly affected by PD along with discrimination/slurs experience.

## Introduction

On March 11, 2011, a 9.0 magnitude earthquake and series of tsunami attacked the northeastern coast of Japan (the Great East Japan Earthquake). Tokyo Electric Company (TEPCO) Fukushima Daiichi Nuclear Power Plant (Daiichi) was heavily damaged, eventually leading to plant explosions, nuclear plant meltdowns, release of radioactive materials, and mandatory evacuation of the surrounding residents. It became the largest nuclear disaster since the 1986 Chernobyl accident, and only the second disaster (along with Chernobyl) to measure Level 7 severity on the International Nuclear Event Scale. Recovery efforts are expected to continue for decades.

Chernobyl mental health studies [1]–[3] suggest that among the affected population, plant workers are at particular risk for experiencing psychological distress. The Fukushima nuclear plant workers have been working under extremely hazardous conditions [4], and a majority of the workers have been under a multitude of stressors. In addition to workplace traumatic stress, such stressors include victim experiences, grief reactions, and the criticism from the public due to their company's post-disaster management [5].

Responses occurring at the time of a trauma and immediately after (i.e., peritraumatic responses) include emotional changes (e.g., helplessness, guilt, horror, and fear of death) and physical reactions (e.g., sweating, shaking, and bladder/bowel responses). A meta-analysis [6] has suggested that such peritraumatic distress (PD) is one of the strongest predictors of future posttraumatic stress responses (PTSR), such as intrusion, avoidance/numbing, and hyperarousal, subsequently developing posttraumatic stress disorder (PTSD) among the affected individuals.

Our previous study [7] examined the mental health outcomes of the Fukushima Daiichi and Daini workers 2–3 months post-disaster. This report suggested their enormous and complex disaster exposures resulted in high rates of general psychological distress and PTSR. As of the writing of this article, little is known about the psychological pathway mechanism from multiple nuclear disaster exposures, PD, to PTSR among the affected people. In order to explore this development pathway of PTSR, we conducted a cross-sectional study to explore this association among Fukushima nuclear plant workers post-accident.

## Methods

Following approvals from the Ethics Committees of Ehime University and National Defense Medical College, full-time TEPCO employees of Fukushima Daiichi and the nearby Daini nuclear power plants (Daiichi: *n* = 1,053; Daini: *n* = 707) were invited to participate in the present study, 2–3 months post-disaster (May–June, 2011). Daini is located 12 km south of Daiichi, had suffered tsunami attacks, and was close to nuclear meltdown. None of the workers had reported acute radiation exposure symptoms. Written consent was obtained from subjects upon enrollment in the study.

We gathered information about respondents' socio-demographic information, disaster-related stressors, and the extent of PD using a self-report questionnaire. Disaster-related stressors were determined based on our initial on-site services [5] and dichotomously coded as “yes” or “no.” We asked subjects whether they had experienced discrimination/slurs (*sabetsu/chuushou* in Japanese) because TEPCO workers were under public criticism. Our studies revealed that PTSR in workers were complex and linked to their multiple disaster experiences, including work-related trauma, disaster victim distress, grief experience, and discrimination from the public [5], [7]. We assessed colleague death(s) as a potential stressor because two young Daiichi employees and a Daini contractor had died due to tsunami.

PD was measured using a Japanese version of the Peritraumatic Distress Inventory (PDI) [8], [9]. The PDI is a 13-item scale quantifying fear and sense of helplessness in the period during and immediately after a traumatic experience. The response format was a five-point Likert scale ranging from 0 to 4; the total score ranged from 0 to 52, and higher scores represent higher PD. A study among motor vehicle accident survivors showed a PDI cutoff score of 22/23 to predict PTSD [10]. The scale's internal consistency is high (Cronbach's alpha = 0.86).

PTSR was quantified using a Japanese version of the Impact of Event Scale-Revised (IES-R) [11]. This is a 22-item scale measuring PTSR domains of intrusion, avoidance/numbing, and hyperarousal. The detailed explanation is available on our previous paper [7].

Among those recruited, 1,495 individuals (Daiichi: *n* = 885, Daini: *n* = 610) participated. PDI scores were missing for 84 subjects; thus, a total of 1,411 subjects (Daiichi, *n* = 831; Daini, *n = *580) were enrolled in the final analysis (response rate: total, 80.2%; Daiichi, 78.9%; Daini, 82.0%).

IBM SPSS Statistics version 22 (IBM Japan, Tokyo, Japan) was used for the statistical analysis. Significance level was set at *p*<0.05 (two-tailed). At the beginning of the analysis, we used chi-square tests to compare differences in subject characteristics between the Daiichi and Daini subgroups. As their features were considerably different, we performed further processes separately for the two groups.

Secondly, we investigated the relationships between independent variables and PD using bivariate regression analysis. In this process and hereafter, categorical variables were handled as continuous variables, ranging from 0 to 1. Significant independent variables were considered potential PDI factors, and they were subsequently entered in the multiple regression analyses.

Thirdly, we examined the relations between PD and PTSR. According to preceding studies [8], [9], we first held a confirmatory factor analysis among the 13 PDI items in order to explore whether or not each item was relevant to IES-R. We subsequently performed bivariate regression analysis to observe the associations between PDI (total score as well as 13 items) and IES-R.

Lastly, we tested the associations between PTSR and independent variables as well as PD. Similar to the previous analyses, we first conducted a bivariate regression analysis, and subsequently multiple regression analysis. Following these calculations, we created pathway maps to test our conceptual model of how independent variables associate with PD and/or PTSR.

## Results

Table 1 shows differences in disaster-related experiences between Daiichi and Daini subjects. Compared with Daini, Daiichi subjects had higher rates of disaster-related experience, except in the areas of discrimination/slurs and family member death(s) experience.

**Table 1:** Comparisons of two subject groups (Daiichi vs. Daini).

			Subject groups							
			Total		Daiichi		Daini		Daiichi vs. Daini	
			*n*	%	*n*	%	*n*	%	?^2^	*p*
**Total**			1,411	100	831	100	580	100		
**Sociodemographic factors**	Age, years	20–29	381	25.6	227	25.7	154	25.4		
		30–39	347	23.3	202	22.9	145	23.9		
		40–49	395	26.5	235	26.6	160	26.4		
		50–59	348	23.4	211	23.9	137	22.6		
		60–69	18	1.2	8	0.9	10	1.7	2.09	0.72
	Sex	Male	1,337	94.8	804	96.8	533	91.9	15.5	<0.001***
	Supervisory work status	Yes	147	10.4	86	10.3	61	10.5	0.07	0.79
	Preexisting illness(es)	Yes	203	14.4	126	15.2	77	13.3	0.96	0.33
**Disaster-related experiences**	Discrimination/slurs	Yes	179	12.7	115	13.8	64	11	2.97	0.085
	Near-death experience	Yes	593	42	446	53.7	147	25.3	117	<0.001***
	Escape from tsunami	Yes	175	12.4	82	9.9	93	16	12.9	<0.001***
	Witnessing of plant explosion(s)	Yes	372	26.4	303	36.5	69	11.9	112	<0.001***
	Family member death(s)	Yes	81	5.7	50	6	31	5.3	0.11	0.74
	Colleague death(s)	Yes	249	17.6	166	20	83	14.3	7.49	0.006**
	Major property loss	Yes	408	28.9	269	32.4	139	24	11.1	0.001**
	Home evacuation	Yes	945	67	582	70	363	62.6	8.2	0.004**

**p<0.01.

***p<0.001.

Table 2 shows the relations between independent variables and PDI. For both groups, PDI associated with multiple disaster exposures (discrimination/slurs, near-death experience, escape from tsunami, witnessing of plant explosion[s], and major property loss). For Daiichi, PDI was related to colleague death(s) experience for Daiichi; female gender, non-supervisory work status, and presence of preexisting illness(es) for Daini.

**Table 2:** Bivariate and multivariate relationships: peritraumatic stress and independent variables.

			Associations with PDI															
			Daiichi (*n = *831)								Daini (*n* = 580)							
			Bivariate analysis				Multivariate analysis				Bivariate analysis				Multivariate analysis			
			B	SE	β	*p*	B	SE	β	*p*	B	SE	β	*p*	B	SE	β	*p*
**Sociodemographic factors**	**Age**	Years	−0.08	0.03	−0.10	0.004**	−0.04	0.03	−0.04	0.24	−0.06	0.03	−0.07	0.077				
	**Sex**	Male	2.93	1.83	0.06	0.11					3.32	1.31	0.11	0.012*	2.96	1.19	0.09	0.013*
	**Supervisory work status**	Yes	−3.16	1.06	−0.10	0.003**	−1.76	1.01	−0.06	0.08	−3.56	1.16	−0.13	0.002**	−2.73	1.06	−0.10	0.010*
	**Preexisting illness(es)**	Yes	0.23	0.91	0.01	0.80					2.02	1.06	0.08	0.056				
**Disaster-related experiences**	**Discrimination/slurs**	Yes	5.58	0.92	0.21	<0.001***	3.61	0.84	0.13	<0.001***	6.59	1.11	0.24	<0.001***	4.38	1.05	0.16	<0.001***
	**Near-death experience**	Yes	7.35	0.60	0.39	<0.001***	5.62	0.61	0.30	<0.001***	6.45	0.78	0.33	<0.001***	4.33	0.80	0.22	<0.001***
	**Escape from tsunami**	Yes	5.92	1.07	0.19	<0.001***	2.19	1.00	0.07	0.028*	5.36	0.95	0.23	<0.001***	2.98	0.93	0.13	0.001**
	**Witnessing of plant explosion(s)**	Yes	4.79	0.65	0.25	<0.001***	2.53	0.63	0.13	<0.001***	4.29	1.10	0.16	<0.001***	2.48	1.01	0.09	0.015*
	**Family member death(s)**	Yes	1.15	1.36	0.03	0.40					1.07	1.60	0.03	0.50				
	**Colleague death(s)**	Yes	3.93	0.80	0.17	<0.001***	1.67	0.78	0.07	0.033*	1.83	1.02	0.07	0.074				
	**Major property loss**	Yes	3.86	0.68	0.19	<0.001***	2.30	0.63	0.12	<0.001***	4.56	0.82	0.23	<0.001***	2.87	0.77	0.14	<0.001***
	**Home evacuation**	Yes	1.97	0.71	0.10	0.005**	1.20	0.64	0.06	0.059	1.38	0.74	0.08	0.062				

*p<0.05.

**p<0.01.

***p<0.001.

Table 3 shows the relations between PD and PTSR. The PDI total score, along with all of the 13 PDI items, associated with IES-R for both groups (*p*<0.001). According to the confirmatory factor analysis, one-factor solution accounted for 38.3% of the total variance. Standardized coefficients of the items were all >0.44, except that of item 9, which was 0.34.

**Table 3:** PDI scores, confirmatory factor analysis of PDI items, and associations with IES-R.

		Total (*n* = 1,411)	Daiichi (*n* = 831)						Daini (*n* = 580)					
		Factor analysis†	Score		Associations with IES-R				Score		Associations with IES-R			
			Mean	SD	B	SE	β	*t*	Mean	SD	B	SE	β	*t*
**PDI total score**			19.46	9.35	1.13	0.05	0.66***	24.9	15.89	8.64	1.13	0.05	0.67***	21.5
**PDI items**	1. I felt helpless to do more	0.69	1.51	1.24	5.79	0.39	0.46***	14.7	1.20	1.17	6.57	0.43	0.54***	15.2
	2. I felt sadness and grief	0.75	2.06	1.29	5.94	0.38	0.48***	15.8	1.81	1.29	5.43	0.41	0.48***	13.3
	3. I felt frustrated or angry I could not do more	0.65	1.77	1.30	5.12	0.38	0.42***	13.3	1.48	1.31	4.50	0.42	0.41***	10.8
	4. I felt afraid for my safety	0.72	1.94	1.35	4.68	0.38	0.40***	12.4	1.39	1.23	4.63	0.45	0.40***	10.4
	5. I felt guilt that more was not done	0.62	1.37	1.24	5.08	0.41	0.40***	12.4	1.04	1.18	5.92	0.44	0.49***	13.4
	6. I felt ashamed of my emotional reactions	0.60	0.70	0.96	7.70	0.51	0.47***	15.1	0.57	0.86	7.32	0.63	0.44***	11.7
	7. I felt worried about the safety of others	0.44	3.21	1.04	3.70	0.53	0.24***	7.00	3.10	1.05	2.36	0.56	0.18***	4.25
	8. I had the feeling I was about to lose control of my emotions	0.68	0.91	1.15	7.07	0.41	0.52***	17.3	0.82	1.09	6.42	0.48	0.49***	13.4
	9. I had difficulty controlling my bowel and bladder	0.34	0.09	0.43	10.2	1.23	0.28***	8.26	0.07	0.33	14.0	1.71	0.32***	8.19
	10. I was horrified by what happened	0.64	2.69	1.30	4.74	0.40	0.39***	12.0	2.51	1.32	3.46	0.43	0.32***	8.08
	11. I had physical reactions like sweating, shaking and pounding heart	0.67	1.06	1.27	6.21	0.38	0.50***	16.5	0.79	1.11	7.26	0.45	0.56***	16.2
	12. I felt I might pass out	0.46	0.28	0.79	7.26	0.65	0.37***	11.2	0.17	0.54	10.39	1.00	0.40***	10.4
	13. I felt I might die	0.64	1.84	1.55	3.82	0.33	0.38***	11.6	0.91	1.26	3.85	0.45	0.34***	8.60

***p<0.001.

† One-factor solution accounted for 38.3% of the total variance.

Table 4 shows the bivariate and multivariate relations between PTSR and independent variables as well as PDI. For both groups, PTSR highly associated with PD (Daiichi: adjusted β, 0.66; *p*<0.001; vs. Daini: adjusted β, 0.67; *p*<0.001). PTSR also associated with discrimination/slurs experience (Daiichi: adjusted β, 0.11; *p*<0.001; vs. Daini, adjusted β, 0.09; *p = *0.005) and presence of preexisting illness(es) (Daiichi: adjusted β, 0.07; *p* = 0.005; vs. Daini: adjusted β, 0.15; *p*<.0001). For Daiichi, PTSR negatively correlated with near-death experience (adjusted β, −0.09; *p* = 0.003); for this variable, Variance Inflation Factor (VIF) was 1.26, and regarding other variables, VIF ranged from 1.01 to 1.31. For Daini, PTSR associated with tsunami escape experience (adjusted β, 0.07; *p* = 0.044). VIF ranged from 1.02 to 1.25.

**Table 4:** Associations between posttraumatic stress responses (IES-R) and independent variables: bivariate and multiple regression analyses.

	Associations with IES-R															
	Daiichi (*n* = 831)								Daini (*n* = 580)							
	Bivariate regression				Multiple regression				Bivariate regression				Multiple regression			
	B	SE	β	*p*	B	SE	β	*p*	B	SE	β	*p*	B	SE	β	*p*
**Sociodemographic factors**																
Age, years	0.02	0.05	0.02	0.68					0.07	0.05	0.06	0.19				
Sex	4.93	2.98	0.06	0.10					6.56	2.12	0.13	0.002**	2.22	1.63	0.04	0.18
Supervisory work status	−1.26	1.86	−0.02	0.50					−1.08	1.95	−0.02	0.580				
Preexisting illness(es)	3.78	1.53	0.09	0.014*	3.23	1.16	0.07	0.005**	7.78	1.73	0.18	<0.001***	6.60	1.33	0.15	<0.001***
**Disaster-related experiences**																
Discrimination/slurs	10.5	1.53	0.23	<0.001***	5.03	1.23	0.11	<0.001***	10.6	1.85	0.23	<0.001***	4.11	1.46	0.09	0.005**
Near-death experience	6.34	1.07	0.20	<0.001***	−2.74	0.93	−0.09	0.003**	7.38	1.33	0.22	<0.001***	−1.31	1.12	−0.04	0.24
Escape from tsunami	7.61	1.78	0.15	<0.001***	2.20	1.42	0.04	0.12	8.23	1.58	0.21	<0.001***	2.59	1.28	0.07	0.044*
Witnessing of plant explosion(s)	4.31	1.12	0.13	<0.001***	−0.85	0.91	−0.03	0.35	3.43	1.83	0.08	0.062				
Family member death(s)	3.44	2.32	0.05	0.14					2.13	2.61	0.03	0.41				
Colleague death(s)	4.50	1.37	0.11	0.001**	0.85	1.07	0.02	0.43	−0.05	1.70	0	0.98				
Major property loss	5.66	1.16	0.17	<0.001***	1.00	0.92	0.03	0.28	6.48	1.36	0.19	<0.001***				
Home evacuation	3.50	1.18	0.10	0.003**	0.87	0.91	0.03	0.34	2.09	1.22	0.07	0.09	0.23	0.92	0.01	0.81
**PDI total score**	1.13	0.05	0.66	<0.001***	1.12	0.05	0.66	<0.001***	1.13	0.05	0.67	<0.001***	1.06	0.06	0.63	<0.001***

*p<0.05.

**p<0.01.

***p<0.001.

**Figure 1:**
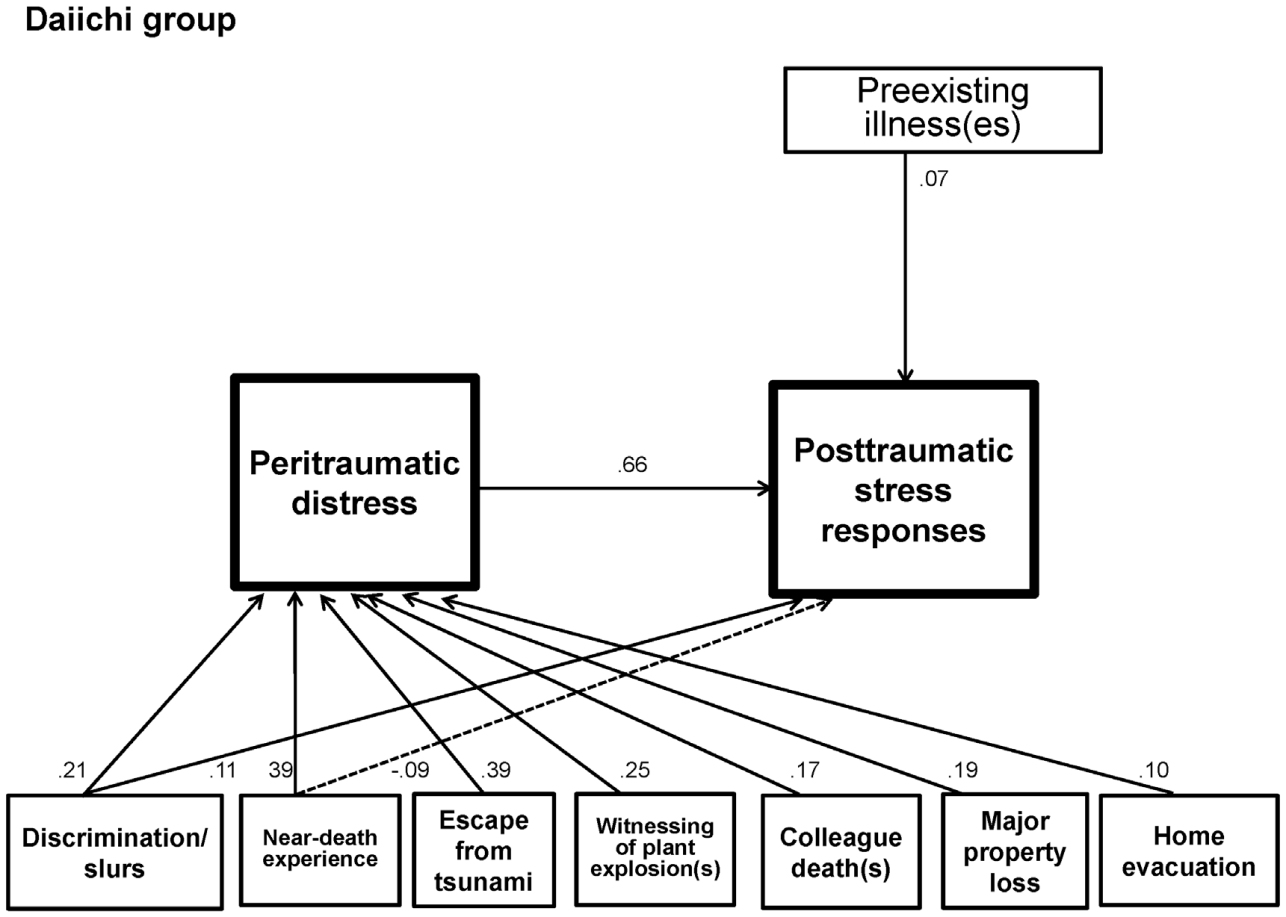
Path model for the posttraumatic stress responses of the Daiichi group. All paths have significance of p<0.05. A dotted arrow shows a negative correlation. PD serves as an intermediary variable between various disaster exposures and PTSR. Discrimination/slurs experience was related to both PD and PTSR, whereas presence of preexisting illness(es) associated solely with PTSR.

**Figure 2:**
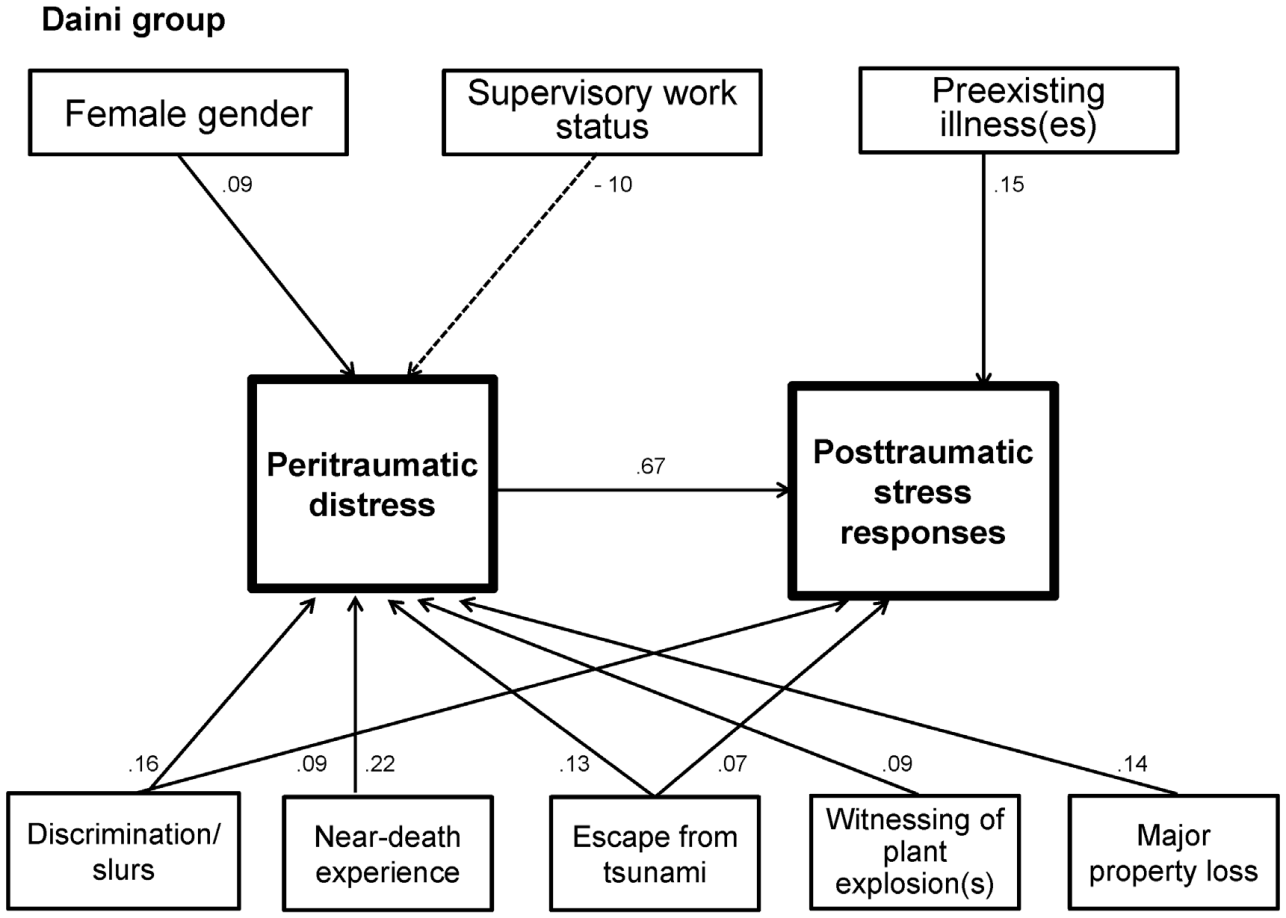
Path model for the posttraumatic stress responses of the Daini group. All paths have significance of p<0.05. A dotted arrow shows a negative correlation. PDI serves as an intermediary variable between various disaster exposures and PTSR. Discrimination/slurs experience was related to both PD and PTSR. Female gender was a risk factor for PD, whereas supervisory work status was a proactive factor. Presence of preexisting illness(es) associated with PTSR but not PD.

Figures 1 and 2 represent the psychological pathway models for the Daiichi and Daini groups, respectively. They show the mechanisms on how socio-demographic factors and various disaster exposures associate with PD and/or PTSR.

## Discussion

To our knowledge, this is the first large-scale study to examine the relationships between disaster-related exposures, PD, and PTSR following a severe nuclear disaster. Overall, Daiichi workers had higher disaster exposures than the Daini workers, and their mechanism path was complex. Our previous study [7] preliminarily reported that discrimination/slurs experience was associated with PTSR for both Daiichi and Daini groups. This paper showed further relationships of discrimination experiences and not only PTSR but also PD. First, this highlights the high impact and complexity of workers' traumatic experiences in this earthquake/tsunami/nuclear disaster. Natural disasters are generally perceived as beyond human control, whereas people tend to believe technology can be controlled, and entrust specific social organizations to do so. Thus, technological disasters have an identifiable responsible party, providing a focus for blame and compensation as well as anger, frustration, fear, and hostility [12]. Given the subjects' public role, criticisms from the very people they had been trying to protect might have an extreme impact on their peritraumatic/posttraumatic mental health. Media communication strategies might be useful for mitigating public responses [13], [14] and follow-up studies will be essential to elucidate these topics.

For both subject groups, various disaster-related exposures associated with higher PD. Our result is consistent with studies of PD among motor vehicle accident survivors [8], [15,], although we need to be cautious about this interpretation due to differences in the nature of the traumatic events. For Daini subjects, non-supervisory work status was associated with high PD, suggesting that a sense of control is an important modulator of risk for posttraumatic outcomes [16]. There was also a relation between PTSR and item 7 of PDI (‘I felt worried about the safety of others’); this trend may be due to their organizational role during the accident. It might be helpful for job supervisors to consider vulnerabilities of workers in non-supervisory positions and emphasize safety issues during recovery efforts.

In our data, PD was a major predictive factor of PTSR. PDI was originally developed by Brunet and colleagues [9] to explore the A2 criterion of PTSD in DSM-IV [17], although there has been discussions on whether or not to utilize it for diagnostic reasons [18], and this criterion was not used as a diagnostic criterion for the DSM5, the revised diagnostic manual [19]. Nonetheless, our results show a strong relation between PD and PTSR, and further studies are essential to better understand these concerns.

It has been demonstrated that women have higher rates of PTSD than men [20]. In this study, we found an association between sex and PD in Daini but not Daiichi. In our sample, over 90% of study subjects were men; therefore, the small sample size of women might have contributed to this result. Future studies should examine sex differences in rates of PD. Experience of family member death(s) also was not relevant to the outcome; this issue warrants further research.

For both groups, individuals with preexisting illness(s) were likely to have higher PTSR, but not PDI. This is consistent with previous studies that those with preexisting medical conditions are vulnerable to post-disaster PTSD [21]. However, our interpretation is limited, as we did not gather diagnostic information about specific illnesses.

This study has various limitations. First, our sample included employees of a single company and, therefore, cannot be generalized to all on-site workers or disaster workers in general. In addition, the use of self-report data from questionnaires may be less accurate than data collected in a face-to-face interview. We also were unable to obtain information about radiation exposure doses as well as other socio-demographic variables (e.g., educational, marital, or socioeconomic status). Finally, our study is cross-sectional and did not measured longitudinal outcomes.

Despite these limitations, our study examined factors related to PD in workers following a large-scale nuclear disaster. Additional studies will be essential for understanding the relationships between PD, PTSR, and long-term psychosocial consequences.
